# The Role of Immune Cells in Oxi-Inflamm-Aging

**DOI:** 10.3390/cells10112974

**Published:** 2021-11-01

**Authors:** Irene Martínez de Toda, Noemi Ceprián, Estefanía Díaz-Del Cerro, Mónica De la Fuente

**Affiliations:** 1 Department of Genetics, Physiology, and Microbiology (Unit of Animal Physiology), Faculty of Biology, Complutense University of Madrid, 28040 Madrid, Spain; nceprian@ucm.es (N.C.); estedi01@ucm.es (E.D.-D.C.); mondelaf@ucm.es (M.D.l.F.); 2 Institute of Investigation 12 de Octubre (i+12), 28041 Madrid, Spain

**Keywords:** aging, immune cells, oxidative stress, inflammatory stress, biological age

## Abstract

Aging is the result of the deterioration of the homeostatic systems (nervous, endocrine, and immune systems), which preserve the organism’s health. We propose that the age-related impairment of these systems is due to the establishment of a chronic oxidative stress situation that leads to low-grade chronic inflammation throughout the immune system’s activity. It is known that the immune system weakens with age, which increases morbidity and mortality. In this context, we describe how the function of immune cells can be used as an indicator of the rate of aging of an individual. In addition to this passive role as a marker, we describe how the immune system can work as a driver of aging by amplifying the oxidative-inflammatory stress associated with aging (oxi-inflamm-aging) and inducing senescence in far tissue cells. Further supporting our theory, we discuss how certain lifestyle conditions (such as social environment, nutrition, or exercise) can have an impact on longevity by affecting the oxidative and inflammatory state of immune cells, regulating immunosenescence and its contribution to oxi-inflamm-aging.

## 1. Introduction

The aging process can have multiple definitions depending on the perspective from which it is considered. From a biological point of view, the aging process may be defined as the progressive and general deterioration of the functions of the organism that leads to a lower ability to react to changes and preserve homeostasis adaptively [1]. Homeostasis includes all processes that organisms use to actively maintain or adjust to appropriate conditions necessary for survival. Thus, although aging should not be considered a disease (it would be absurd to think of an illness that affects 100% of people), it is the main risk factor for the occurrence of chronic age-related diseases [2]. There are three physiological systems, the nervous, endocrine, and immune systems, in charge of maintaining body homeostasis. Moreover, these systems are in continuous communication, constituting a neuroimmunoendocrine system, which allows the preservation of homeostasis and, therefore, of health [3]. With aging, there is a functional decline of these homeostatic systems and an impairment in the communication between them [2,4], which translates into a worse capacity to mount an adequate response to a variety of stressors. The decay of this capacity, which has also been referred to as decreased homeodynamic space [5,6] or decreased homeostatic resilience [1], is what results in higher morbidity and mortality. Nevertheless, the age-related changes in these homeostatic systems are established at different rates in each subject, which translates into a different rate of aging or biological age of individuals with identical chronological age [2,4].

We believe that the rate at which these homeostatic systems decline relies on the establishment of a chronic oxidative and inflammatory stress situation. Thus, we describe how the oxidation inflammation theory of aging (oxi-inflamm-aging) is one of the most complete to describe how the process of aging occurs. Even though we are aware that the aging process is multifactorial, we propose mitochondrial reactive oxygen species (ROS) production as the first event involved in this process. In addition, we provide molecular mechanisms that link oxidation and inflammation and demonstrate how immune cells play an essential role in interconnecting both processes and, consequently, modulating the rate of aging. Accordingly, we show how the function and redox state of immune cells can be used as markers of the rate of aging of an individual allowing the prediction of lifespan. Moreover, to further confirm the role of immune cells in the aging process, we show, by modulating the redox and inflammatory state of immune cells and the production of oxidant and pro-inflammatory compounds by these cells, how different situations or conditions, such as the social environment, nutrition, and exercise, can have an impact on the lifespan of the organism.

## 2. Following the Free Radical and Mitochondrial Theory of Aging

Many theories were proposed to explain how the process of aging occurs. Among them, the free radical theory of aging proposed by Harman [7] and further developed by several authors [8,9,10,11] is probably the most widely accepted one. This epigenetic theory proposes that aging is the consequence of damage accumulation by deleterious oxidation of biomolecules caused by the high reactivity of the free radicals and reactive oxygen species (ROS) produced in our cells because of the necessary use of oxygen. Oxygen (O_2_) is essential for the synthesis of adenosine triphosphate (ATP) in the mitochondrial respiratory chain, which is believed to be the primary site of ROS production, acting as the final acceptor of four electrons, generating one molecule of water. However, when the reduction in oxygen is not full, reactive oxygen species are generated. Thus, when oxygen captures one electron, the superoxide anion (O_2_^−^) is formed, which can lead to hydrogen peroxide (H_2_O_2_) and hydroxyl radical (OH^−^). O_2_^−^ and OH^−^ are free radicals given that they have an unpaired electron, which makes them highly reactive towards all biomolecules, whereas H_2_O_2_ is not. Even though H_2_O_2_ is not a free radical, it can result in OH^−^ being considered as an important oxidant. Hence, the term reactive oxygen species (ROS) is generally used to include them all. These ROS act as second messengers and coordinate several molecular pathways within the cells [12]. Nevertheless, they have to be quickly neutralized by antioxidant defenses to avoid the generation of oxidative damage to the different cellular components. Thus, all aerobic organisms have developed antioxidant defenses, both enzymatic and non-enzymatic, to keep these ROS between appropriate ranges. However, with aging, there is an imbalance between oxidant and antioxidant compounds in favor of the former due to uncontrolled production of oxidants and/or due to a decrease in antioxidant defenses, which generates what is known as an oxidative stress situation. The establishment of oxidative stress exposes cells to a pro-oxidant environment that entails the accumulation of damage of the different biomolecules (proteins, lipids and, nucleic acids), loss of function, and cell death.

The free radical theory of aging has been criticized by several authors, doubting its usefulness to explain how the aging process occurs, indicating that oxidative damage does not represent the cause of aging [13,14]. For example, Gladyshev [13] concludes that the role of ROS in aging is not universal, with the idea that aging still occurs under anaerobic conditions in yeast cells. However, it is not correct to apply the idea of aging to unicellular organisms since aging should be understood in the physiological context of pluricellular animals with sexual reproduction. Some other concerns that were put forward against the role of ROS in aging were based on some works in which increased oxidative stress has led to increased longevity [15,16]. These results, however, far from dismantling the free radical theory of aging, can be explained due to a hormetic response. Thus, a short-term increase in ROS production can cause an adaptive response by increasing antioxidant expression [17,18,19], whereas chronic ROS levels beyond a certain threshold are still damaging for cellular components. Other claims against the free radical theory of aging are some studies in which the use of antioxidants did not increase longevity in mammals, as some authors stated [14]. Nevertheless, confusion between maximum and mean longevity is one of the reasons for this criticism. Actually, species with higher longevity have fewer antioxidants because they do not need them since they produce a lower amount of ROS [20]. In addition, there are some other examples in which ingestion of a diet enriched in antioxidants increases longevity, as will be discussed in the last section of this review. Another argument against the theory is the fact that overexpression of antioxidant enzymes does not extend the lifespan of invertebrates (*Drosophila melanogaster*) and mammals (*Mus musculus*) [21,22]. However, caution should be taken when interpreting the causes of aging by the use of genetically manipulated animals, as they can develop other adaptive mechanisms to counteract a specific mutation. Interestingly, a 20% increase in lifespan was observed when upregulation of catalase expression was targeted to the mitochondria specifically [23].

## 3. mitROS, the First in the Aging Process

The mitochondrial free radical theory of aging proposed that the mitochondrial rate of ROS (mitROS) production is the most relevant fact in the aging process [2,8,11,20]. In the cell, there are other sources of ROS, such as the endoplasmic reticulum (ER), nucleus, peroxisomes, and even the Golgi apparatus. The production of ROS through the NADPH oxidases (NOX) family, considered a major source of ROS in eukaryotic cells, in the membranes of these compartments, and the cytoplasmatic membranes of the cells are higher than in the mitochondria [24,25]. NOX family constitutes the only professional primary oxidases since other enzymes such as, for example, xanthine oxidase (XO) or monoamine oxidases (MAO) produce ROS as a consequence of their primary metabolic function [26]. Thus, the NOX-related ROS production in the cytoplasmatic membrane, principally that from innate immune cells using NOX-2, represents an important source of extracellular ROS [27]. Nevertheless, mitochondria, despite their relatively low, well-controlled, and regulated rate of ROS generation, can be the most relevant for aging. The reason behind this, suggested previously, is the presence of DNA in this organelle. The mitochondrial DNA (mtDNA) can be easily damaged by mitROS present in its vicinity because it lacks histones, which makes it more vulnerable to oxidative damage [28]. This damage to mtDNA alters mitochondria homeostasis and the function of these organelles impairing cell function; the fact that it is more relevant in postmitotic cells that cannot fully regenerate mitochondria, where it was suggested that the aging process starts [2]. Moreover, recent evidence has shown that oxidative damage to mtDNA generates mtDNA fragments that can travel to other organelles, such as to the nucleus, and insert into the nuclear DNA (nDNA), amplifying the damage around the cell [29]. In order to support this idea, it was shown that the accumulation of mtDNA fragments in nDNA increases with aging, and it is reversed by rapamycin, a treatment that increases longevity [30]. In addition, these fragments of mtDNA and even those of nDNA can be released outside the cell to extracellular fluids and recirculate into the bloodstream, reaching other locations far from the tissue in which they were produced [31,32] and triggering the activation of immune cells, as is commented in the next section.

For all aforementioned, we consider that this oxidative stress is the first cause of aging, and this idea has universal application, one of the conditions that any theory that tries to explain the cause of aging must have. This age-related oxidation occurs at the different levels of biological organization (molecular, cellular, tissue, and whole-organismal level) in all multicellular animals, including human subjects [2]. For this reason, we suggest that this oxidative stress should be considered as the basis of the nine hallmarks of aging [33]. In fact, genomic instability, telomere attrition, epigenetic alterations, loss of proteostasis, deregulated nutrient-sensing, mitochondrial dysfunction, cellular senescence, stem cell exhaustion, and altered intercellular communication can have as an origin mitROS production and the consequent establishment of oxidative stress.

## 4. Oxidation and Inflammation, Always Together. Oxi-Inflamm-Aging

Even though inflamm-aging is not included as one of the nine hallmarks of aging, possible because these hallmarks were focused on markers within the cells, there is a universal agreement that aging is accompanied by the establishment of a low-grade chronic inflammation at the systemic level [34]. This chronic inflammatory stress is established when there is an imbalance between pro-inflammatory compounds and anti-inflammatory compounds in favor of the former. It is known that immune cells need to produce pro-inflammatory mediators to carry out their defensive functions. Thus, inflammation is not a negative phenomenon per se since it is needed to maintain life through a constant struggle to preserve the integrity of the individuals [34,35]. However, this response has to be tightly regulated and finished shortly after the resolution of the noxious agent, which is mainly achieved by the triggering of an anti-inflammatory response by immune cells. Nevertheless, as we age, this transient inflammatory process becomes chronic [34,35]. It was suggested that this could happen due to the persistence of the antigenic challenge or weakening of the regulatory systems of the immune response [36]. We propose that this chronic inflammatory stress can be the result of the establishment of chronic oxidative stress by the immune system’s activity. Currently, it is clear that oxidation and inflammation are linked processes since excessive or uncontrolled free radical production can induce an inflammatory response, and free radicals are inflammatory effectors [35]. Indeed, both oxidation and inflammation occur when the immune system responds to the invasion of pathogens. This chronic inflammation is characterized by mononuclear immune cell infiltration (monocytes, macrophages, and lymphocytes) to different tissues where these cells produce excessive ROS and pro-inflammatory mediators to conclude this situation but, at the same time, generate tissue damage and fibrosis. Therefore, a continued and active oxidant response by immune cells can lead to cellular damage due to ROS overproduction, which can also recruit other inflammatory cells leading to additional pro-inflammatory and oxidant production amplifying cellular damage [37,38]. Different pathways were proposed to mediate the connection between both inflammation and oxidation (reviewed in [4,35]). Continuing with the idea that mitROS production is the first event in the aging process, it was demonstrated that mtDNA fragments generated due to continued ROS leakage in the mitochondria over time act as danger or damage-associated molecular patterns (DAMPs) that can bind to pattern recognition receptors (PRR) and through activation of the nuclear transcription factor kappa b (NF-κB) could activate the expression of pro-inflammatory cytokines, boosting inflamm-aging [39,40]. Moreover, it is also known that mitROS can activate NACHT, LRR, and PYD domains containing protein 3 (NLRP3) inflammasome, which leads to the processing and secretion of the pro-inflammatory cytokines interleukin-1 and 18 [41,42,43,44].

Based on this link between oxidation and inflammation, the oxidative-inflammatory theory of aging emerged [2] to provide a more complete and integrative vision of the most relevant processes involved in the aging process. Thus, aging would be the consequence of chronic oxidative stress, associated with inflammatory stress, which would cause the deterioration of the function of all cells of the individual, but would have a greater impact on those of the homeostatic systems, that is, the nervous, endocrine, and immune systems, which would explain the lower ability to maintain homeostasis that occurs with aging and leads to increased morbidity and mortality. Furthermore, this theory introduced the involvement of the immune system in the greater or lesser oxidation and inflammation that appears as we age. Since immune cells need to produce oxidant and inflammatory compounds to carry out their defensive function, when uncontrolled, they may be responsible for the generation of oxidative-inflammatory stress that would not only cause their functional deterioration (immunosenescence) but could also increase these stresses in the body, accelerating the aging process. Given that phagocytes (neutrophils in humans and macrophages in mice) are the main immune cell type that generates oxidants throughout the “respiratory burst” in which NADPH oxidase and xanthine oxidase enzymes participate, they were proposed to play a central role in oxi-inflamm-aging [2,45].

## 5. Impact of Immunosenescence in Oxi-Inflamm-Aging

The establishment of a chronic oxidative stress situation, as the basis of aging, occurs in all types of cells of the body. However, in the cells of the immune system, as it is one of the main regulatory systems, this fact translates into a much wider variety of damage around the whole body. Given the main role of the immune system as a homeostatic system, all the changes and reorganizations that immune cells experience with age or immunosenescence underpins poorer responses to vaccination, lower capacity to mediate anti-cancer responses, more oxidation and inflammation, accumulation of senescent cells and tissue damage, along with autoimmunity and loss of control of persistent infections [46,47]. These changes can be divided into those affecting the abundance of different subpopulations and those influencing the functional capacity of these cells. Concerning the first ones, immunosenescence has been linked to an inverted ratio CD4/CD8 and with the accumulation of T memory cells, among many others, which were recently reviewed [47,48,49]. These changes are thought to be the result of the immunological history of the individual and, as such, an adaptation to the circumstances in the old [46] (i.e., the higher need of T CD8 cytotoxic cells due to increased cancerous cells, or no need to maintain the T naïve cell repertoire, given the little chance of discovering a new antigen at the very old age). However, although these changes could be adaptive in this sense, at the same time can be detrimental, as the drop in regulatory CD4 T cells can cause some acute responses not being able to be terminated on time and keep causing oxidation and inflammation. In this context, it was also proposed that the increase in one subpopulation can be a compensatory mechanism due to a reduced function of the cell; thus, if these cells are working less, a higher number of these cells will be required, as it was described that it occurs in natural killer cells with aging [50,51]. More recently, some other studies found that the function of a specific cell subpopulation can change in the elderly, as is the case of the CD4 T cells in supercentenarians that were found to become cytotoxic [52].

Based on the above, to truly evaluate which age-related changes in the immune system can have an impact on the aging rate, it becomes apparent that the study of specific functional capacities of immune cells, rather than existing subpopulations, becomes a better choice. In this sense, immunosenescence causes a higher adherence of neutrophils and leukocytes to endothelium, hindering their migration to potential points of infection, together with a lower chemotaxis capacity to move towards these points [53,54]. The impairment of these functions is thought to be caused by increased oxidative stress within these cells and at the endothelial level, which stimulates the production of integrins and cadherins, which enhances their attachment to the endothelium and makes their migrating abilities difficult [55], a fact that translates into a higher incidence of infection. Another function that is diminished with age is the phagocytic capacity of neutrophils and macrophages [53,54]. This decreased phagocytic ability results in the persistence of oxidative and inflammatory neutrophils in damaged sites, which may contribute to a failure in the mechanisms that promote the resolution of inflammation, ultimately leading to tissue damage and even mortality [56,57]. In addition, tissue-resident macrophages are also key in the clearance of other senescent cells [58,59]. Thus, on the one hand, the diminished phagocytic ability of these cells would make us prone to infectious agents, but at the same time, it could cause tissue damage and accumulation of apoptotic and senescent cells within our body, contributing to persistent activation of immune cells and more oxidation and inflammation [49]. Something similar occurs with respect to the proliferation of T lymphocytes in response to a mitogen, which was shown to decrease with age [54]. Proliferation, activation, and secretion of cytokines by T cells are regulated by intracellular ROS, which play a fundamental role in peripheral T cell homeostasis [60]. However, excessive and prolonged exposure to high ROS concentrations induces immune dysfunction, inhibiting T cell proliferation and leading to apoptosis [61]. The natural killer activity, which is the capacity that immune cells have to destroy a cancerous cell, as well as virus-infected cells, dampens with age, and it is one of the parameters that has been mostly related to the susceptibility to suffer infections and with increased mortality in old age, both in humans and mice [62,63]. NK cell-mediated clearance of senescent cells is an essential aspect of tissue homeostasis [64] and tumor growth limitation [65], and as such, the age-related diminished NK cell function results in an accelerated accumulation of senescent cells in various tissues [59,66] and to uncontrolled malignant cell division [67].

**Figure 1:**
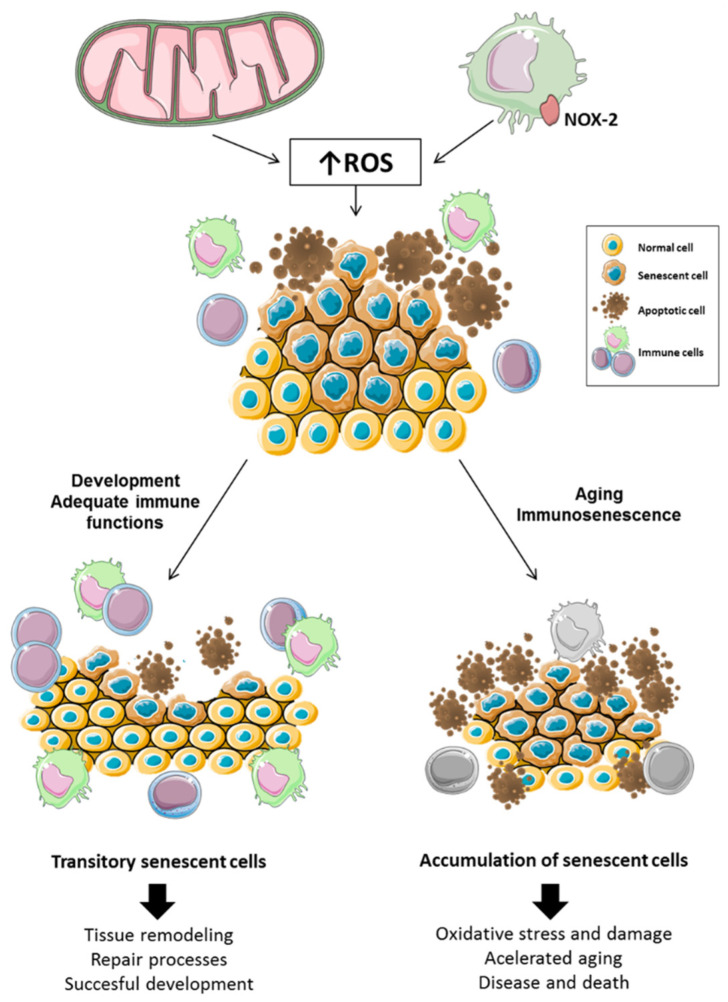
**Influence of immunosenescence in the accumulation of senescent cells.** Oxidative compounds come mainly from two sources, the mitochondria and NOX-2, located in the membrane of immune cells, especially in phagocytes. These oxidative compounds lead to senescent and apoptotic cells. However, senescence can be transient and homeostatic or chronic and damaging depending on the efficiency of immune cells in removing them. Thus, from the fetal stage to adulthood, the senescent cells that appear are transitory thanks to the adequate function of immune cells. Thus, immune cells remove these cells, promoting regeneration and repair processes, which are crucial for proper development. However, from adulthood to death, chronic exposure to ROS produces oxidative tissue damage and deterioration of the function of immune cells (immunosenescence), which causes accumulation of senescent and apoptotic cells, leading to accelerated aging and, consequently, increased morbidity and mortality.

In this regard, immunosenescence was suggested to underlie the accumulation of senescent cells that occur with aging. Cellular senescence, although often used as a synonym of aging, has a homeostatic and regulatory role promoting clearance of damaged and potentially cancerous cells as well as coordinating wound healing and tissue repair, among other processes. Thus, when cells are exposed to an excess of ROS, to prevent malignant transformation, they can enter a senescence-associated secretory phenotype (SASP). Through SASP, they release pro-inflammatory mediators to attract immune cells such as macrophages, natural killer cells, or cytotoxic T cells, which recognize and eliminate those non-functional or malignant cells [68], promoting body homeostasis. However, the age-related decline in the function of immune cells causes senescent cells to accumulate chronically, which causes damage to the organism by promoting inflammation, tumorigenesis, and tissue dysfunction [69,70]. Thus, it is the age-related deterioration of the immune system that causes senescence to switch from a temporal and homeostatic process to a chronic and damaging situation [71], an idea that is illustrated in Figure 1.

**Figure 2:**
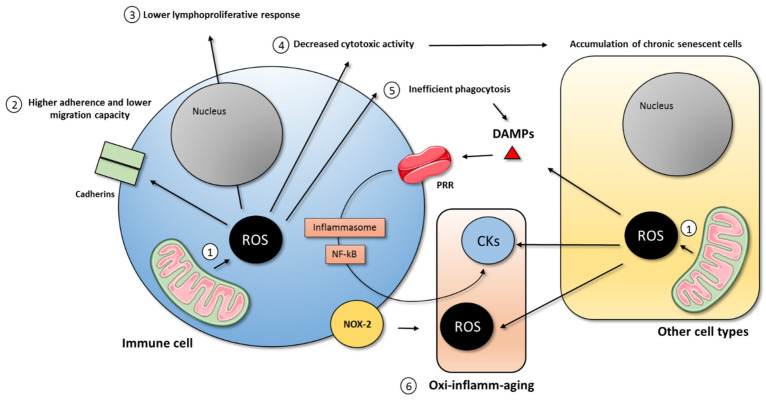
**Effects of immunosenescence in oxi-inflamm-aging**. 1. Aging starts with the leakage of reactive oxygen species (ROS) that happens in the mitochondria, that react with the mitochondrial DNA (mtDNA) and cause mitochondrial dysfunction, which increases ROS production and generation of mtDNA fragments. These fragments travel to the nucleus and insert into nDNA generating DNA damage and genomic instability. These ROS beyond a threshold cause the cell to enter senescence and develop a senescence-associated secretory phenotype (SASP), which happens in both immune and non-immune cell types. 2. ROS impact the expression of cadherins and integrins, causing a higher adherence and lower migration capacity of immune cells. 3. DNA damage results in a lower lymphoproliferative response. 4. ROS decrease the cytotoxic activity of immune cells that is responsible for the accumulation of chronic senescent cells in other tissues. 5. Uncontrolled ROS balance also leads to inefficient phagocytosis, which results in the increased concentration of several molecules from both immune and non-immune cells that behave as damage-associated molecular patterns (DAMPs) and that can bind to immune cells and throughout inflammasome and nuclear factor kappa B (NF-κB) activation induce the production of inflammatory mediators (cytokines, CKs) causing inflamm-aging. The accumulation of chronic senescent cells, together with the increased pro-inflammatory mediators at the systemic level, causes excessive production of ROS by immune cells, mainly phagocytes through NADPH-oxidase (NOX-2), to destroy and remove these senescent cells. 6. This situation generates vicious spiral feedback of oxidation and inflammation with aging (oxi-inflamm-aging), in which immune cells play a central role.

In addition, the age-related loss of the tightly regulated function of immune cells results in the altered production of pro- and anti-inflammatory mediators. In light of this, immunosenescence was also proposed to be responsible for inflamm-aging, both directly and indirectly [47,72]. The direct form is that by their ability to produce inflammatory mediators, when the defensive functions are not tightly regulated, it can result in the accumulation of pro-inflammatory mediators at the systemic levels. Even though several cell types contribute to the age-related low grade of chronic inflammation throughout SASP, it was proposed that senescent macrophages are a key driver of inflamm-aging [73,74]. Moreover, damaged and dying cells release endogenous molecules called damage-associated molecular patterns (DAMPs), which analogously activate the immune system to pathogen-associated molecular patterns (PAMPs) to fight the pathogen or resolve the damage in this case. These DAMPs bind to pattern recognition receptors (PRR), which activate the transcription factor NF-κB and the inflammasome pathways resulting in the sustained production of pro-inflammatory compounds, together with oxidant compounds, causing tissue damage, cell senescence, and the release of DAMPs in vicious spiral feedback [47,72]. This idea is illustrated in Figure 2. Further demonstrating the role that NF-κB activation has in the rate of aging, we demonstrated that those mice that had the lowest activation of NF-κB at old age were the ones that lived longer [75]. In addition, supporting the role of mitochondrial ROS in aging, it was recently demonstrated that making dysfunctional mitochondria only in T cells induces multimorbidity and premature aging in mice [76]. Thus, immunosenescence, the aging of the immune system, is a result of the aging process, but it also acts as a driver of this process, producing oxidant and inflammatory compounds which cause damage and induce senescence within other tissues.

## 6. Can Immunosenescence Be a Marker of the Rate of Aging in Each Individual?

As was previously mentioned, the age-related decline that the homeostatic systems undergo does not take place at the same rate in a group of individuals of the same species and the exact chronological age. This fact led to the concept of biological age, which means the real rate of aging of an individual. However, several different parameters were proposed as biomarkers of biological age (telomere length [77], DNA methylation [78], plasma proteome profile [79], among others; we focus on those involving the function and redox state of the immune cells.

In order to be a marker of the rate of aging, the values of a given parameter have to be related to the lifespan of an individual. Thus, our research group focused on finding which of the age-related changes in the functions exerted by immune cells can be related to the longevity of mice. In this context, we first demonstrated that prematurely aging mice that have an inadequate response to stress show at the adult age immune function parameters and oxidative stress parameters closer to old animals, and they have a shorter lifespan [54,80]. Afterward, a battery of immune function and oxidative stress parameters were analyzed in adult mice and then left to naturally age until death, and the individual lifespan of each mouse was written down. Throughout multiple linear regression, we were able to develop mathematical models for lifespan prediction based on the values of immune function and oxidative stress parameters that mice showed at the adult age [81], demonstrating that both the function and oxidative stress of immune cells from mice at the adult age relate to their lifespan. With these results, and with the previous investigations showing that the age-related changes in the function and oxidative stress parameters follow similar patterns in mice and humans [54,80], we focused on developing a mathematical model throughout multiple linear regression for estimation of the biological age based on the immune cells’ function, which we called the ImmunolAge, in humans, with the Immunity Clock [82]. The Immunity Clock encompasses five immune function variables: neutrophil chemotaxis and phagocytosis abilities, lymphocyte chemotaxis and proliferation abilities, as well as cytotoxic natural killer activity. Based on the status of these immune functions in an individual, we can estimate their aging rate. Accordingly, we demonstrated that women with anxiety, as is further discussed in the next section, have a higher ImmunolAge than their chronological age, which means that they are aging at a faster rate. On the opposite side, we found out that centenarians exhibited a lower ImmunolAge than their chronological age, which confirms the idea of extremely long-lived people aging at a slower rate.

With respect to oxidative stress parameters, we established a Redox signature of Aging and Longevity throughout principal components analysis, by which in a 2D-graph we were able to differentiate age groups based on their antioxidant and oxidant markers [80]. While adult, mature and elderly were different groups, nonagenarians showed overlapping areas with adult signatures, suggesting that a controlled redox balance underlies healthy aging. Centenarians, in this case, were characterized by the highest antioxidant capacities, which could indicate that at this age, they need this high antioxidant component to maintain appropriate redox balance or that only those that can express high antioxidant defenses are those that reach high longevity.

In this context, some other research groups also proposed other markers of immunosenescence to estimate the rate of aging. For example, Alpert and collaborators proposed an IMM-AGE score [83] based on different immune subsets frequency dynamics through aging, and it was found to predict mortality better than other aging clocks based on epigenetic parameters such as DNA methylation, i.e., Epigenetic Clock [78]. More recently, Sayed and colleagues proposed an inflammatory clock of aging (iAge) developed by machine learning based on soluble markers of chronic inflammation [84]. Altogether, our results and others demonstrate that a small battery of immune function and redox parameters of immune cells, as well as inflammatory markers, can be useful for the determination of the aging rate of an individual, that is, the quantification of their biological age.

## 7. Lifestyle Situations Modulating the Rate of Aging: Friends or Foes?

As was previously mentioned, the maintenance of homeostasis at the physiological level and, therefore, of the health of individuals is ensured by the tightly regulated interplay of the three homeostatic systems: the nervous, immune, and endocrine systems. These systems are constantly exchanging information throughout neurotransmitters, hormones, and cytokines, and it is of such importance that it is known as neuroimmunoendocrine communication. As living organisms, we are continuously exposed to and adapting to different stressors. The stress response can be defined as the adaptive physiological modifications that occur as the consequence of any internal or external changes or threats (stressors). Thus, an individual reacts to a physical or mental stressor that is potentially health-threatening by activating interconnected neuroimmunoendocrine circuits. This pro-survival response allows the body to face and deal with the challenge and re-establish homeostatic equilibrium, promoting health. However, if the individual perceives a noxious stimulus as too intense, or its duration as too long, they may fail to cope with it, maladaptation occurs, neuroimmunoendocrine parameters remain altered, accelerating the rate of aging and the appearance of age-related diseases [4,85,86]. In accordance with this, there are several examples in which chronic stress situations were associated with a defective immune functional capacity [87,88,89] and with accelerated aging [4,90]. In addition, our research group has a wide experience analyzing the relationship between the response to a stressful situation and longevity in mice. We have demonstrated that those mice that have an inappropriate response to stress when exposed to the behavioral T-test have higher oxidant and inflammatory compounds in their immune cells [80,91]; they consequently show premature immunosenescence [92,93] and frailty [94], and shorter lifespan than those that react “normally” to the stressful situation [54,80,94].

**Figure 3:**
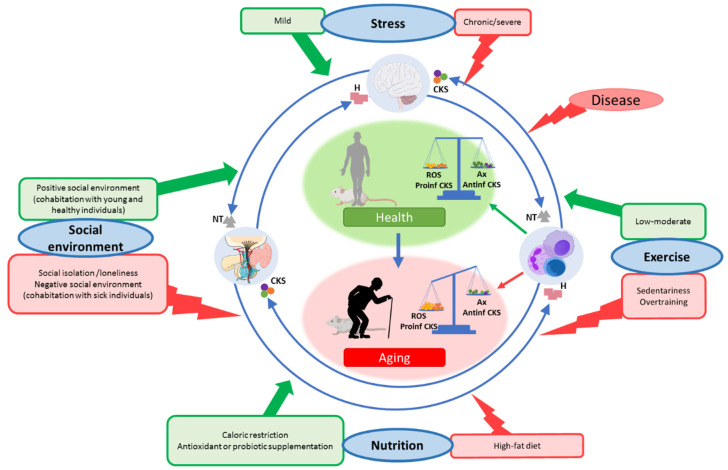
**Dual effect of different lifestyle situations on oxi-inflamm-aging**. The communication between the regulatory systems (nervous, endocrine, and immune systems), known as neuroimmunoendocrine communication, ensures the homeostasis of the organism and, therefore, health. This crosstalk takes place by the existence of receptors in each homeostatic system for neurotransmitters (NT), hormones (H), and cytokines (CKS). Oxi-inflamm-aging alters the function of the regulatory systems as well as the neuroimmunoendocrine communication, which causes age-related increased morbidity and mortality. External factors, such as the social environment, nutrition, and exercise, can modify the oxidative and inflammatory stress of the organism and the function of immune, nervous, and endocrine cells, altering this communication, and consequently, modulating the aging rate of an individual. Thus, a negative social environment, which can be the isolation of an individual (rodents) or loneliness (humans), or the cohabitation with sick or older subjects, affects the neuroimmunoendocrine communication negatively, increasing the rate of aging while a positive social environment, understood as living with healthy or younger individuals, enhances this communication, decreasing the aging rate. With respect to nutrition, high-fat diets and obesity, as well as an excess of antioxidant supplementation, accelerate the rate of aging, whereas caloric restriction or supplementation with adequate amounts of vitamins, antioxidants, or probiotics decelerate it. Finally, whereas a sedentary life or overtraining can negatively impact the aging rate, low and moderate exercise was shown to slow down aging. All these conditions modulate the function of immune cells by increasing or decreasing oxidant, pro-inflammatory, antioxidant, and anti-inflammatory compounds; therefore, they can alter the rate of oxi-inflamm-aging. Antinf CKS: anti-inflamatory cytokines; Ax: antioxidants; CKS: cytokines; H: hormones; NT: neurotransmitters; Proinf CKS: pro-inflammatory cytokines.

In the context of the stress response, the dose or intensity of the stressor is an important fact, given that exposure to mild stresses relates to better health, whereas severe stress is associated with impaired health. Exposure to mild stresses over time can have a beneficial impact on the health of an organism by triggering counteracting mechanisms that over time translates into a strengthened resistance capacity, a concept that was referred to as “hormesis” [95]. In this sense, exposure to a short-term variety of stressors was proposed as an efficient intervention to promote health [95,96]. In this context, we discuss how some lifestyle conditions can be, at the two ends of a spectrum, both beneficial or detrimental, focusing on how they affect the oxidative and inflammatory stress and the function of the immune system, and consequently modulating lifespan. All these ideas are illustrated in Figure 3.

### 7.1. Social Environment

In social species, such as humans and rodents, the social context is essential for survival and reproductive success as it provides protection from environmental threats. According to this, several studies linked social isolation in mice [97,98] and loneliness in humans [99,100] or living with sick mates [101] with a suppression of both the innate and adaptive immune response [89,102] throughout an increase in oxidative [103,104] and inflammatory [105,106] compounds. Unsurprisingly, lonely and socially isolated individuals were also found to die earlier than their more socially integrated counterparts [107].

However, at the other end of the spectrum, the existence of positive, strong social networks was positively associated with health [108,109]. Recently, we demonstrated that cohabitation of old mice with adult ones for two months could improve immune function through the modulation of redox and inflammatory states of peritoneal immune cells from old mice, which translates into these animals having a longer lifespan than those who cohabitated only with old mice [110]. Moreover, this cohabitation strategy was similarly performed with adult prematurely aging mice (PAM) and non-prematurely aging ones, obtaining a significant decrease in oxidation and inflammation, improving the immune function, and increasing the longevity of PAM [111]. These results suggest that an adequate social environment, possibly by increasing social communication, strengthening social bonds, and reducing the stress associated with the age-related increase in loneliness and social distance [112], is an effective strategy to delay oxi-inflamm-aging and consequently achieve a longer lifespan. This fact is of great importance given the COVID-19 situation and the associated social environment restrictions.

### 7.2. Nutrition Conditions

The deep impact that nutrition has on our health is widely accepted [113,114]. On the one hand, inadequate nutrition is known to worsen the health status [115,116,117,118]. Accordingly, diets rich in fat are associated with obesity and with the establishment of both oxidative and inflammatory stresses [119,120,121,122]. This inflammation, caused by the pro-inflammatory signals released from adipocytes (adipokines), leads to an altered immune function [121,122] and immune cell populations [123,124]. All of which, in turn, accelerate oxi-inflamm-aging and, consequently, decrease the lifespan [121,122].

On the other hand, nutrition can be a useful intervention to delay aging and treat aging-related diseases [125,126]. These interventions include caloric restriction [127,128,129,130,131], variations in macronutrient ratios [132,133], dietary supplementation with probiotics [134,135,136], vitamins [137,138] and antioxidants [139,140], among many others. Various pieces of evidence demonstrate that all these types of nutritional interventions can modulate immune function in aging [138,139,141,142,143,144,145], in addition to improving the oxidative and inflammatory state of these cells [136,138,139,144,146], thus slowing down oxi-inflamm-aging and lengthening life expectancy [147,148,149]. In particular, we performed several antioxidant nutritional strategies and demonstrated that they could ameliorate oxidative stress in immune cells, improving the function of these cells, diminishing oxidative-inflammatory stress in humans [138,150,151,152] and in mice in which we verified that these changes translate into an increased lifespan [139,142,153].

Similarly, supplementation with probiotics was also proposed to exert its beneficial effects through immunomodulation. Given the brain-gut-microbiota axis and the shared molecules by which these organs communicate, probiotic supplementation can reverse the oxidative and inflammatory stress that is established in immune cells with aging [136,154,155,156], improving the function of these cells [136,144,154,157,158,159] and, consequently, increasing lifespan [160,161,162].

### 7.3. Physical Exercise

The impact that physical exercise has on health was widely studied. During physical exercise, it is known that reactive oxygen species (ROS) are increased both in the skeletal muscle and at the systemic level [163]. However, depending on the intensity and duration of this exercise, this fact can be detrimental or can cause a beneficial effect in the long term. Thus, excessive exercise or overtraining promote oxidative [164,165] and inflammatory stress [166,167], which causes immunosuppression [168,169,170,171,172,173] and, consequently, they could accelerate the rate of aging of an individual and the appearance of age-related diseases.

Nevertheless, if the physical exercise is of moderate intensity and performed regularly for transient periods, the induced ROS production plays a role in the induction of antioxidants, DNA repair, and protein degrading enzymes, resulting in a better redox balance and delaying the aging process [174,175,176,177]. In fact, in elderly individuals, moderate exercise leads to clear immune system benefits such as T-cell function, antibody production, macrophage responses, cytokine modulation, and naïve/memory cells ratio, among others [178,179,180,181], and, consequently, it decreases the susceptibility to infectious processes and increases longevity in both rodents and humans [182]. The described effects on immune cells could be achieved by the modulation of antioxidant and anti-inflammatory mechanisms, increasing the level of anti-inflammatory cytokines (e.g., Type 2 helper T, IL-10, and IL-4) and decreasing pro-inflammatory compounds levels (e.g., number of inflammatory CD14+CD16+ monocytes, TNF-α, pro-inflammatory adipokines, IL-6, HSPs) [181,183,184,185]. Moreover, it is known that regular and mild exercise mitigates mitochondrial aging and interrupts the vicious cycle of oxidative damage [181], which also explains its anti-aging properties.

### 7.4. Age-Related Diseases

Until now, we discussed the effect that different conditions have on the aging rate of individuals through modulation of oxidative and inflammatory states and the function of immune cells. Furthermore, it is known that most, if not all, age-related diseases such as diabetes, cardiovascular disease, neurodegenerative diseases, chronic renal disease, and cancer, among others, [2,186,187,188,189,190,191,192] are associated with increased oxidative and inflammatory stress. Moreover, in all of them, the function of immune cells is altered, which has made several authors consider the patients of these pathologies to suffer accelerated aging [193]. Given that all these age-related diseases share a common denominator, which is age, by focusing on controlling or delaying the aging process for the establishment of a chronic oxidative and inflammatory stress situation, we would also manage to delay the appearance of all the above-mentioned diseases, which is much more effective than individually trying to target each one of them.

## 8. Conclusions

We propose that aging, understood as the general deterioration of the homeostatic systems, is the consequence of the establishment of a chronic oxidative and inflammatory stress situation (oxi-inflamm-aging) that dampens the function of all cells in the organism. However, the functional decline of the homeostatic systems, namely the nervous, endocrine, and immune systems, as well as the impairment of the neuroimmunoendocrine communication, is the cause of the age-related increased morbidity and mortality. Thus, the function of cells from these systems could be used to estimate the rate of aging of an individual. We propose the use of immune cells as markers of health, above those from the other homeostatic systems, given that immune cells can be easily obtained and studied without sacrificing the individual. Moreover, given the close crosstalk that these systems keep among themselves (the so-called neuroimmunoendocrine communication), the state of immune cells also reflects how the other homeostatic systems are. Furthermore, given that immune cells circulate throughout the body, their state could reflect that of other tissues. In addition to being an indicator of the rate of aging of each individual, we suggest that the immune system is also a driver of aging. This hypothesis is based on the fact that the age-related decline in the function of immune cells (immunosenescence) amplifies the oxidative and inflammatory damage of the organism by their uncontrolled production of ROS and pro-inflammatory mediators and by the diminished capacity of removing senescent cells from around the body.

Taking all this into consideration, by modulating the function of immune cells, we can modulate the rate of aging and the lifespan of an individual. We demonstrate that this can be accomplished with easy and affordable lifestyle strategies, such as social environment, nutritional interventions, and exercise. We believe that these conditions affect the rate of aging by their antioxidant and anti-inflammatory actions; nevertheless, more research is needed to unravel the specific underlying mechanisms. We consider that the study of long-lived individuals can help us to shed light on the aging process by identifying which molecular pathways they activate or repress at a given time point in their aging process that allows them to live beyond the average lifespan of the species. Thus, future research should be aimed at disentangling those mechanisms to slow aging and reach healthy longevity.

Although in this review, we propose a possible sequence of events that would explain the impairment of the immune system and its impact on the general aging of the individual, further research should confirm our proposal and investigate what makes the switch from processes that have allowed each individual to complete development in adult age (strong immune system, ROS, pro-inflammatory mediators, senescent cells) be drivers of aging after adulthood when the aging process starts.

## Data Availability

Not applicable.
